# The effect of confounding variables on the relationship between anthropometric and physiological features in 2000-m rowing ergometer performance

**DOI:** 10.3389/fphys.2023.1195641

**Published:** 2023-05-30

**Authors:** Robert Podstawski, Krzysztof Borysławski, Zoltán Alföldi, Ihász Ferenc, Jacek Wąsik

**Affiliations:** ^1^ Department of Physiotherapy, University of Warmia and Mazury in Olsztyn, Olsztyn, Poland; ^2^ Institute of Health, Angelus Silesius University of Applied Sciences, Wałbrzych, Poland; ^3^ Doctoral School of Health Sciences, Faculty of Health Sciences, University of Pécs, Pécs, Hungary; ^4^ Department of Sport Sciences, Faculty of Education and Psychology, Eötvös Loránd University, Szombathely, Hungary; ^5^ Department Kinesiology and Health Prevention, Jan Dlugosz University in Czestochowa, Czestochowa, Poland

**Keywords:** rowing performance, different age, somatic features, physiological parameters, sex categorization

## Abstract

**Background:** Anthropometric and physiological characteristics are often considered as potential variables that are significantly related with motor performance.

**Aim:** The aim of this study was to identify and weigh the key anthropometric and physiological characteristics that are associated with 2000-m rowing ergometer performance in male and female athletes.

**Methods:** The study involved 70 best female and 130 best male rowers from the seven largest Hungarian rowing clubs, classified into one of the following age categories: juniors (36 women and 55 men, age range: 15-16 years), older juniors (26 women and 52 men, age range: 17-18 years), and seniors (8 women and 23 men, age range: over 18 years). Anthropometric and body composition measurements were determined by the bioelectrical impedance method proposed by Weiner and Lourie (1969), and skin fold measurements were conducted to estimate relative body fat content. The countermovement jump test and the 2000-m maximal rowing ergometer test were used for physiological measurements.

**Results:** An increase in skeletal muscle mass was correlated (*r* = -.39, *p* <.001) with a significant decrease in rowing time over a distance of 2000 m, whereas a significant increase in rowing time was noted with an increase in sitting height (only in men, *r* = .33, *p* <.001), body mass (in women and men: *r* = .24, *p* = .013 and *r* = .31, *p* = .009), and body fat percentage (*r* = .26, *p* < .030). Rowing time was also bound by a significant correlation with maximal force (*r* = -.79 and -.90, *p* <.001) and relative maximal power (*r* = -.54 and -.78, *p* <.001) in both sexes, with relative peak power in males (*r* = -.51, *p* < .001), and with estimated relative maximal aerobic capacity in females (*r* = -.43, *p* <.001).

**Conclusion:** Rowing performance over a distance of 2000 m is significantly negatively correlated with the skeletal muscle mass, maximal force, relative maximal power, relative peak power, and estimated relative maximal aerobic capacity.

## Introduction

Athletes participating in various sports differ in anthropometric and physiological characteristics (APCh) (Stoop et al., 2018; Lau et al., 2020). The performance and APCh of male and female rowers have been studied extensively among both juniors (Bourgois et al., 2000; Claessens et al., 2002; Claessens et al., 2005; Alacid et al., 2011; Giroux et al., 2017) and seniors (Hebbelinck et al., 1980; Jürimäe et al., 2000; Battista et al., 2007; Forjasz, 2011; Durkalec-Michalski et al., 2019; Penichet-Tomás et al., 2019), although significantly more studies have been conducted on male than female rowers. Some researchers compared the APCh of rowers to the general population (Malina, 1994; Shephard, 1998; Bourgois et al., 2000; Schranz et al., 2010). Several studies analyzed the APCh of rowers from different age and rank groups (Mikulić, 2008; Klusiewicz et al., 2014; Podstawski et al., 2022), and they posited that this approach facilitates the selection of individuals with a predisposition for rowing (Alfoldi et al., 2021). However, there is a general scarcity of published studies on sexual dimorphism in rowing (Keenan et al., 2018; Podstawski et al., 2022).

The combination of both APCh is a good predictor of rowing performance (Jurimae and Jurimae, 2002), although some traits may play a greater role in rowing efficiency. Rowing is a strength endurance sport, where body size and body mass undoubtedly affect performance (Secher and Vaage, 1983; Jürimäe et al., 2010). According to Battista et al. (2007) and Penichet-Tomás et al. (2019), fat-free body mass, which is related primarily to skeletal muscle mass (SMM), is undoubtedly the key predictor of rowing performance, which could be attributed to the fact that high-performance rowers engage around 70% of all muscles when rowing a boat (Richter et al., 2010). Anthropometric studies also agree on the importance of body height as a determinant of rowing performance (Forijasz, 2011; Akça, 2014). The laws of physics and biomechanics play a key role in all sports that use oars, and they reveal the possibilities and limitations of the human body at the physical and anthropometric level (Penichet-Tomás et al., 2019). According to Shimoda et al. (2009), stroke power consistency contributes to the maintenance of power during ergometer rowing. However, opinions are divided on the extent to which various parts of the body contribute to rowing. According to Crosgrove et al. (1999), successful elite rowers generate around 75%–80% of power with the lower limbs and around 20%–25% with the arms during the rowing stroke. As regards the involvement of different muscle groups (such as antagonistic muscles) in rowing performance, rowers use mainly extensor muscles which, if lower limb muscles are inadequately trained, generate the force necessary to propel the boat (Ogurkowska et al., 2015). This is probably because that the increase in torso torque is much smaller in extensor muscles than flexor muscles (Podgórski et al., 2020). Therefore, considering both classifications, especially in sliding seat rowing, including the Olympic modality, lower limbs are the most important part of human body used in rowing compared to other rowing categories (Kollias et al., 2004), and the stroke range is greater in rowers with longer limbs (Claessens et al., 2005; Rakovac et al., 2011). Physiological and biomechanical characteristics undoubtedly influence rowing performance (Richter et al., 2010; De Larochelambert et al., 2020), and some of them play a significantly more important role and can be regarded as the main factors.

As regards physiological characteristics, aerobic metabolism covers 75%–80% of the demand for energy in a 2000-m rowing race (Jurišic et al., 2014). In view of the above, the anaerobic threshold and maximal oxygen uptake (VO_2max_) are most frequently used to diagnose, program, and control the training process in rowing (Mikulić, 2008; Klusiewicz et al., 2014; Martin and Tomescu. 2017). High anaerobic capacity is also crucial for high-level performance (Mäestu et al., 2005).

Models of body constitution and the physiological profiles of competitors participating in various competitive events have been described extensively in the literature (Bourgois et al., 2000; Fell and Gaffney, 2001; Claessens et al., 2002; Jürimäe and Jürimäe, 2002; Claessens et al., 2005; Slater et al., 2006; Mikulic, 2008; Forjasz and Kuchnio, 2009; Forjasz, 2011) and validated in studies of young elite rowers. However, to the best of our knowledge, very few studies have investigated anthropometric and physiological determinants together with the rowing pace in athletes aspiring to become elite rowers. A research methodology combining APCh with racing pace determinants could expand our understanding of rowing performance for the needs of the selection process. Therefore, the aim of this study was to identify and indicate anthropometric and physiological characteristics that are associated with 2000-m rowing ergometer performance in male and female athletes.

## Materials and methods

### Participants

Seventy best female and 130 best male rowers from the seven largest Hungarian rowing clubs participated in this study, which was conducted at Gyor rowing club over a period of four consecutive days in the middle of the racing season (10 days after one rowing regatta and 6 days before the following rowing regatta). The rowers regularly competed at both national and (in some cases) international events. The vast majority of the surveyed subjects were Hungarian Rowing Championship medalists in different categories. Rowers with an international ranking had won medals at European Championship regattas or had qualified for the finals in such regattas. The following inclusion criteria were used in the targeted sampling procedure in all age groups: rowers held a valid competition license and had participated in national and/or international competitions for at least 1 year. All rowers had valid medical certificates. They trained regularly, and their physical activity levels were not limited (for whatever reason) to the extent that could significantly affect their motor fitness. The training program was consistent with the training plan recommended by the Hungarian Rowing Federation: 12–13 h/week for 15- to 16-year-olds, 14–15 h/week for 17- to 18-year-olds, and 16–17 h/week for 19- to 22-year-olds. The aerobic-to-anaerobic training ratio in the above groups was 80:20%, 75:25%, and 70:30%, respectively. In addition, athletes who had received points for participating in international events attended training camps organized by the Hungarian Rowing Federation twice or three times a year (depending on the age group). Each rower was classified into one of the three age categories: juniors (36 women and 55 men, age range: 15–16 years), older juniors (26 women and 52 men, age range: 17–18 years), and seniors (8 women and 23 men, age range: over 18 years). In the senior group, both male and female athletes were relatively young, and the oldest rowers were only 22 years old. The trial was performed in compliance with the guidelines and policies of the Health Science Council, the Hungarian Scientific and Research Ethics Committee (IV/3067-3/2021/EKU), and the Declaration of Helsinki.

Each participant was provided with detailed information about the purpose of the study, potential risks, measurement methods, and motor test techniques that could be practiced during training sessions held directly before the study. All rowers gave voluntary informed consent to participate in the study by signing consent forms.

### Procedures, data collection and equipment

At the beginning of the 2022 racing season, all participants attended anthropometric and physiological tests which were performed in the morning. The rowers were informed that they could have a light meal (800–1,200 kcals) consisting mainly of carbohydrates (60%–70%), but no later than 3–4 h before the trial (Williams, 1999). Anthropometric measurements were performed on days 1 and 2; the athletes participated in motor tests on day 3, and they rowed a distance of 2000 m on day 4. The rowers’ coaches in the sports clubs were instructed not to engage the subjects in any strenuous training the day before the trial, and they assisted the authors in performing the measurements. Body height was measured to the nearest 1 mm with a calibrated Soehlne Electronic Height Rod 5,003 (Soehlne Professional, Germany), in accordance with the relevant guidelines. Body mass (measured to the nearest 0.1 kg), BMI and body composition characteristics, including body fat percentage (BFP) and SMM, were determined by bioelectrical impedance with the InBody 720 (Biospace Co. Inc., Seoul, South Korea) Bioelectrical Impedance Analyzer (BIA). This foot-to-foot, hand-to-hand and hand-to-foot contact device features two stainless-steel foot pad electrodes mounted on a platform scale and a tetrapolar 8-point tactile electrode system with two stainless-steel handles. The reliability of bioelectrical-impedance analysis relative to other body composition measurement methods, such as DXA, has been successfully demonstrated (Sun et al., 2005; Gibson et al., 2008; Shafer et al., 2009). The platform scale uses a single load cell to measure body mass (and stature) and calculate the body mass index (BMI). The body fat percentage (PBF) is determined by summing the results of the segmental lean analysis to determine total lean body mass, fat mass, and the proportion of fat mass to total body mass. The muscle mass percentage (M%) is estimated by evaluating water content in the segmental regions with the use of the provided equations. The visceral fat area (VFA) is estimated with the use of regression equations (provided) which, according to the manufacturer, have been derived from a comparison of visceral fat in computerized tomography scans and impedance in the torso region in a segmental lean analysis. The remaining anthropometric characteristics, such as sitting height [cm], arm span [cm], limb length [cm], and BSA [m^2^], were measured using the methods proposed by Weiner and Lourie (1969). Skin fold measurements (biceps, triceps, scapula, suprailiac, abdomen, thigh, lower leg) were performed using a Harpenden caliper.

#### Estimation of relative body fat content

Relative body fat content was measured with a caliper based on the method developed by Pařízková (1961). According to this procedure, the thickness of five skinfolds is measured (over the biceps and triceps, subscapular, suprailiac and medial calf), and the sum of the obtained values is multiplied by 2; the product is then used to find the estimated relative body fat content in a table.

#### Countermovement jumping

The power output of the lower extremities and the height attained by the center of body mass during vertical jumps were measured with a PJS-4P60S force plate (JBA Zb. Staniak, Poland) with a 400 Hz sampling rate (Gajewski et al., 2018; Batra et al., 2021). The force plate was connected via an analog-to-digital converter to a PC with MVJ v.3.4 software (JBA Zb. Staniak, Poland). The amplifier was connected to a PC via an A/D converter. Measurements were performed using the MVJ v. 3.4 software package (JBA Zb. Staniak, Poland). The calculations relied on a physical model where the participants’ body mass served as a point that is affected by the vertical components of two external forces, namely the force of gravity acting on the body, and the platform’s reactive force. Each subject performed three countermovement jumps (CMJ) with maximal force. A CMJ is a vertical jump from a standing erect position, preceded by a counter movement of the upper limbs and lowering of the body mass center before take-off. Each participant was asked to perform a CMJ from the force plate to determine the maximal force [N] and the rate of displacement [m/s]. Jump height [cm] and peak power [W] were determined based on the above measurements. Jump height was calculated from ground reaction forces. Relative peak power [W/kg] was calculated based on each subject’s body mass.

#### 2000-m maximal rowing ergometer test

The participants were asked to perform an all-out 2000-m test on a certified rowing ergometer (Concept 2 D). The ergometer screen was set to display the number of meters remaining, the average 500-m time, and the accumulated time. Power output in watts (W) was measured over 2000 m. Watts were calculated in a two-step procedure. In the first step, distance was defined as (time/number of strokes) × 500. In the next step, a “split” was defined as 500 × (time/distance). Watts were calculated as 2.8/(split/500). There were minor differences in intensity due to individual variations in stroke value and the ability to keep the 500 m split time constant. Prior to each test, the participants performed a 6-min warm-up over 500 m, followed by a 6-min rest that involved stretching exercises. The estimated relative aerobic capacity (ErVO_2_) was calculated based on the formula developed by McArdle et al. (2006) for men: ErVO2 = (Y × 1,000)/BM, where BM is body mass, and Y = [BM < 75kg; 15.1- (1.5 × time)]; BM => 75 kg; 15.7- (1.5 × time)]. The power delivered over 2000 m was divided by body mass to calculate relative performance (rW 2k).

Neither heart rate (HR) nor acid-base balance indicators, such as the blood concentrations of lactic acid, alkaline deficiency or excess, blood pH, and current molecular pressure of CO_2_, were measured in this study due to time and logistic constraints. A relatively high number of separate measurements had to be performed over three consecutive days to minimize interference with the athletes’ training program and changes in their overall body condition.

### Statistical analysis

The results were processed statistically using Statistica PL v. 13.5 software. *n*-order parametric partial correlation coefficients were calculated. A partial correlation is a correlation between a pair of variables that accounts for their relationships with another (third) variable (first-order partial correlation) or several *n*) other variables (*n-*order partial correlation). This approach is applied to determine whether variables A and B are still correlated when the relationships between the remaining variables are eliminated (which corresponds to the assumption that the remaining variables have constant values). The level of *p* ≤ 0.005 was assumed as statistically significant values of the correlation coefficient. All examined and correlated features had a normal distribution, which was checked with the Shapiro-Wilk test.

A post-hoc power analysis was also performed for partial correlations in IBM SPSS Statistics for Windows, Version 28.0 (IBM Corp. Released 2021. Armonk, NY: IBM Corp.). The sample size is usually selected and the power analysis is usually conducted before data collection, but in the present study, this was not an intentional approach because the entire “population” of rowers who were eligible and available for the study was examined. Therefore, post-hoc power was calculated based on the sample size for the results of the partial correlation analysis. The calculated power denotes the sensitivity of the analysis. The observed power was calculated based on the size of male (*n* = 130) and female (*n* = 70) populations in a two-sided analysis, with an alternative hypothesis of 0.4 at *α* = 0.05 and *α* = 0.01. Depending on the type of the analysis, 3 to 8 partialled variables were eliminated (Table 1). The analysis had very high power in all cases.

**TABLE 1:** Post-hoc power analysis for partial correlations in male and female populations.

Number of partialled variables	Post hoc power estimation			
	Males (*n* = 130)		Females (*n* = 70)	
	*α* = 0.05	*α* = 0.01	*α* = 0.05	*α* = 0.01
3	0.997	0.985	0.927	0.799
4	0.997	0.984	0.923	0.792
5	0.997	0.983	0.920	0.784
6	0.997	0.982	0.915	0.776
7	0.997	0.981	0.911	0.768
8	0.996	0.981	0.907	0.759

## Results

### Analysis 1: Anthropometric characteristics

Only the parameters that were significantly correlated with the time taken to complete a distance of 2000 m on the rowing ergometer were used in the analysis. Skeletal muscle mass [%], which was not significantly correlated in males or females, and BFP, which was correlated only in males, were also considered (Table 2). Interestingly, none of the skinfold measurements were significantly correlated with the time taken to complete a distance of 2000 m in males or females. In both genders, rowing time (p-value ranged from .007 to .001) was significantly negative correlated with an age (r = −.66 and −.57) and anthropometric characteristics such as body height (r = −.70 and −.48), sitting height (r = −.67 and −.38), body mass (r = −.79 and −.68), BMI (r = −.57 and −.46, arm span (r = −.68 and r = −.51), and lower limb length (r = −.47 and −.35). In males, the time taken to complete a distance of 2000 m decreased significantly with a rise in BFP values (r = −.29, *p* = .002).

**TABLE 2:** Raw correlation coefficients between age and the studied anthropometric characteristics vs. the time taken to complete a distance of 2000 m on a rowing ergometer.

Parameter	Male (N = 130)		Female (N = 70)	
	*r*	*P*	*r*	*p*
Age [years]	−.66	<0.001	−.57	<0.001
Body height [cm]	−.70	<0.001	−.48	<0.001
Sitting height [cm]	−.67	<0.001	−.38	0.003
Body mass [kg]	−.79	<0.001	−.68	<0.001
BFP [%]	−.29	0.002	−.18	ns
SMM [%]	.06	ns	.11	ns
BMI [kg/m^2^]	−.57	<0.001	−.46	<0.001
Arm span [cm]	−.68	<0.001	−.51	<0.001
Lower limb length [cm]	−.47	<0.001	−.35	0.007

Notes: ns, not statistically significant.

In most cases, anthropometric parameters and age were significantly positively correlated, which could considerably affect the real correlations between these factors and rowing time over a distance of 2000 m (Table 3).

**TABLE 3:** Correlation coefficients between age and the studied anthropometric characteristics in males and females (values significant at *p* < 0.05 are marked in bold).

Male	[1]	[2]	[3]	[4]	[5]	[6]	[7]	[8]	[9]
Female									
[1] Age [years]		**.35**	**.40**	**.51**	**.30**	.01	**.42**	**.35**	.17
[2] Body height [cm]	**.29**		**.79**	**.68**	.10	−.01	**.21**	**.89**	**.82**
[3] Sitting height [cm]	.25	**.83**		**.63**	**.19**	−.06	**.31**	**.67**	**.50**
[4] Body mass [kg]	**.47**	**.67**	**.64**		**.56**	**−.28**	**.86**	**.61**	**.42**
[5] BFP [%]	.25	−.11	.03	**.43**		**−.76**	**.67**	.09	−.09
[6] SMM [%]	−.10	.09	−.02	**−.41**	**−.92**		**−.36**	.03	.12
[7] BMI [kg/m^2^]	**.37**	−.06	.06	**.70**	**.68**	**−.50**		**.19**	−.02
[8] Arm span [cm]	**.33**	**.91**	**.74**	**.71**	.01	.00	.08		**.78**
[9] Lower limb length [cm]	.10	**.85**	**.68**	**.54**	−.06	−.01	−.09	**.79**	

For this reason, partial correlation coefficients were calculated to control for the values of the remaining attributes (Table 4). The correlations between anthropometric parameters and the time taken to complete a distance of 2000 m on a rowing ergometer changed completely in both genders when the effects of the remaining variables were eliminated. In males, rowing time over a distance of 2000 m was significantly negatively correlated with SMM only (r = −.39, *p* < .001)—an increase in SMM was associated with shorter rowing time. Rowing time over a distance of 2000 m was significantly positively correlated with sitting height (r = .33, *p* < .001) and body mass (r = .24, *p* = .013)—an increase in the above anthropometric characteristics was associated with a significant increase in rowing time over a distance of 2000 m. In females, rowing time was significantly positively correlated with body mass (r = .31, *p* = .009) and BFP (r = .26, *p* < .030) only, which implies that an increase in the above anthropometric characteristics prolonged the time taken to complete 2000 m on the rowing ergometer. In males, rowing time was also significantly shortened with an increase in age.

**TABLE 4:** Further-order partial correlations coefficients between the studied anthropometric characteristics and the time taken to complete a distance of 2000 m.

Characteristics	Male (N = 130)		Female (N = 70)	
	*r*	*p*	*r*	*p*
Age [years]	−.34	<.001	−.03	ns
Body height [cm]	−.06	ns	.11	ns
Sitting height [cm]	.33	<.001	−.04	ns
Body mass [kg]	.24	.013	.31	.009
BFP [%]	.16	ns	.26	.030
SMM [%]	−.39	<.001	−.04	ns
BMI [kg/m^2^]	−.18	ns	−.01	ns
Arm span [cm]	−.01	ns	.03	ns

### Analysis 2: Physiological characteristics

All measured physiological characteristics were considered in the analysis. In males, all physiological characteristics were bound by highly significant negative correlations with rowing time over a distance of 2000 m. In females, rowing time was significantly negatively correlated with only five physical characteristics (Power _peak_, RPP, ERG_rel_VO_2max_, ERGVO_2max_, Force _max_), whereas no significant correlations were noted for jump height, speed max, or RPM (Table 5). In both genders, the time taken to complete 2000 m on a rowing ergometer was significantly (*p* < .001) shortened with an increase in Power_peak_ (r = −.98 and-.99), RPP (r = −.77 and −.76), ERG_rel_VO_2max_ (r = −.56 and −.82), ERGVO_2max_ (r = −.99) and force_max_ (r = −.59 and −.52). In males, rowing time was also significantly shortened with an increase in jump height (r = −.36, *p* < .001), speed _max_ (r = −.34, *p* < .001), and RP_max_ (r = −.27, *p* = .003).

**TABLE 5:** Raw correlation coefficients between age and the studied physiological characteristics vs. the time taken to complete a distance of 2000 m.

Characteristics	Male (N = 130)		Female (N = 70)	
	*r*	*p*	*r*	*p*
Power_peak_ [W]	−.98	<.001	−.99	<.001
RPP [W/kg]	−.77	<.001	−.76	<.001
ERG_rel_ VO_2max_ [mL/kg/min]	−.56	<.001	−.82	<.001
ERG VO_2max_ [L/min]	−.99	<.001	−.99	<.001
Jump height [cm]	−.36	<.001	.02	ns
Speed _max_ [m/s]	−.34	<.001	.01	ns
Force _max_ [N]	−.59	<.001	−.52	<.001
RP_max_ [W/kg]	−.27	.003	.12	ns

Note: RPP, relative peak power; ERG_rel_VO_2max_, estimated relative maximal aerobic capacity; ERG VO_2max_, absolute maximal aerobic capacity; RP_max_, relative maximal power.

In most cases, significant positive correlations were also observed between physiological characteristics (in particular in men), which could affect the real correlations between these attributes and the time taken to complete a distance of 2000 m (Table 6).

**TABLE 6:** Correlation coefficients between age and the studied physiological characteristics in males and females (values significant at *p* < 0.05 are marked in bold).

Male female	[1]	[2]	[3]	[4]	[5]	[6]	[7]	[8]
[1] Power_peak_ [W]		**.78**	**.50**	**.97**	**.34**	**.32**	**.59**	**.25**
[2] RPP [W/kg]	**.75**		**.90**	**.79**	**.35**	**.34**	.14	**.28**
[3] ERG rel VO_2max_ [mL/kg/min]	**.78**	**.97**		**.59**	**.29**	**.30**	−.08	**.27**
[4] ERG VO_2max_ [L/min]	**.99**	**.76**	**.82**		**.35**	**.33**	**.56**	**.27**
[5] Jump height [cm]	−.02	.16	.14	−.02		**.98**	**.18**	**.82**
[6] Speed _max_ [m/s]	−.01	.20	.16	−.01	**.98**		**.19**	**.87**
[7] Force _max_ [N]	**.51**	.03	.13	**.52**	.07	.06		**.36**
[8] RP_max_ [W/kg]	−.13	.10	.08	−.12	**.90**	**.91**	.17	

Therefore, partial correlation coefficients were calculated to control for the values of the remaining attributes (Table 7). It should be stressed that Power_peak_ [W] and ERG VO_2max_ [L/min] were not considered in the analysis because they were significantly correlated (*r* approximated 1.0) with rowing time over a distance of 2000 m. The above observation can be attributed to the fact that Power_peak_ and ERG VO_2max_ values were estimated based on rowing time over a distance of 2000 m (in particular ERG VO_2max_) and were highly correlated (Table 5). The calculated partial correlations would be completely different if these variables were included in the analysis. The correlations between the analyzed parameters and the time taken to complete a distance of 2000 m on a rowing ergometer changed completely in both genders when the effects of the remaining variables were eliminated. In both sexes (more so in women), rowing time over a distance of 2000 m was most significantly correlated with an increase in force _max_ (r = −.79 and −.90, *p* < .001) and RP_max_ (r = −.54 and −.78, *p* < .001). In addition, the time needed to complete 2000 m on a rowing ergometer was significantly shortened with an increase in RPP in males (r = −.51, *p* < .001), and with an increase in ERG_rel_ VO_2max_ in females (r = −.43, *p* < .001).

**TABLE 7:** Further-order partial correlations coefficients between the studied physiological characteristics and the time taken to complete a distance of 2000 m.

Characteristics	Male (N = 130)		Female (N = 70)	
	*r*	*p*	*r*	*p*
RPP [W/kg]	−.51	<.001	−.23	ns
ERG _rel_ VO_2max_ [mL/kg/min]	−.08	ns	−.43	<.001
Jump height [cm]	−.06	ns	−.17	ns
Speed max [m/s]	−.17	ns	−.15	ns
Force max [N]	−.79	<.001	−.90	<.001
RP_max_ [W/kg]	−.54	<.001	−.78	<.001

## Discussion

The present study involved an original approach to interpreting the relationships between 2000-m rowing performance and APCh. The main limitation in research examining the correlations between APCh and motor performance stems from the fact that all variables are related to a certain degree, and the final result in a motor test is affected by numerous factors. The characteristics that significantly correlate with performance during a 2000-m rowing test are even more difficult to identify in young athletes who aspire to become elite rowers. The male and female rowers examined in this study were relatively young, and some of them had already achieved success in European regattas (including European championship), but none had participated in international championships or the Olympics. Young rowers aspiring to become elite athletes constitute a less homogeneous group than, for example, the finalists of the most prestigious regattas, and their performance can also be affected by factors that are unrelated to training (e.g., socioeconomic factors) (Alföldi et al., 2021; Podstawski et al., 2022). Such athletes train in different rowing clubs under the supervision of various trainers, which can also significantly affect their psychomotor development (Shipherd et al., 2019). Moreover, the influence of developmental factors is difficult to eliminate in rowers younger than 18, because the central and peripheral nervous systems are not yet fully anatomically and functionally developed at that age, and a certain imbalance can exist between excitatory and inhibitory neurons (van de Lagemaat et al., 2014; Tornero-Aguilera et al., 2022). Therefore, the procedure of selecting the most promising rowers is highly complex (Podstawski et al., 2022). During training practice, coaches play a key role in identifying the most talented athletes, and their judgement is often regarded as the “gold standard” against which scientific methods of talent identification are compared (Roberts et al., 2021).

In the present study, many variables were strongly correlated with the time of completing the 2000-m rowing performance test; therefore, factors that directly affect performance in rowing, a hybrid effort that requires high levels of both strength and endurance abilities, were difficult to identify (Lawton et al., 2013). The factors that exert the greatest impact on motor performance had been first identified in the 3-Minute Burpee Test (3-MBT) which evaluates endurance-strength abilities (Podstawski et al., 2016a; Podstawski et al., 2016b). In the group of the analyzed variables, only physical activity levels significantly correlated with the strength and endurance of moderately physically active young women and men during the 3-MBT (Borysławski et al., 2020). Despite the fact that 3-MBT and the rowing ergometer test involve similar types of effort, the results of these tests cannot be compared directly because the evaluated rowers, despite their relatively young age, are a more homogeneous and better trained group than university students. Rowers participate in strenuous training programs, most of which are developed for teams and groups in preparation for competitive events, and they are characterized by high and very similar PA levels (Mikulić, 2008; Alfoldi et al., 2021).

### Anthropometric characteristics

In the current study, an increase in SMM in males was associated with a shorter rowing time over a distance of 2000 m. In contrast, greater sitting height and body mass were associated with a significant increase in rowing time over the same distance. The observation that greater body mass and sitting height can compromise rowing performance may seem surprising, as it is generally believed that heavier and taller individuals perform better in this sports discipline (Russel et al., 1998; Forjasz, 2011; Lawton et al., 2013). Despite the fact that sitting height is a component of body height, junior rowers had lower sitting height relative to stature (51.6%) and greater leg length relative to stature (48.4%) in comparison with the normative reference group (52.1% and 47.9% respectively) (Bourgois et al., 2000). Long lower limbs mainly increase the drive phase of the rowing stroke and generate the greatest power during rowing (Secher and Vaage, 1983). In females, higher body mass and BFP prolonged the time taken to complete 2000 m on the rowing ergometer. These results are consistent with the observations made by Battista et al. (2007) and Penichet-Tomás et al. (2019) who found that an increase in SMM with a simultaneous decrease in BFP are needed to improve 2000-m rowing performance. The significance of low BFP in conjunction with increased muscle mass had been previously emphasized in research conducted on elite and sub-elite juniors and seniors (Russel et al., 1998; Cosgrove et al., 1999; Yoshiga et al., 2000; Mikulić, 2008). Rowing is a hybrid sport that requires high strength and endurance abilities, which is why low BFP is essential for high-level rowing performance. Moreover, greater fat-free mass is associated with higher aerobic capacity which is crucial for successful rowing performance (Yoshiga and Higuchi, 2003). In studies designed to determine the optimal predictors of athletic performance (Ingham et al., 2002; Riechman et al., 2002), fat-free mass emerged as one of the strongest predictors of performance when anthropometric characteristics were measured. Therefore, long-term rowing-specific training programs combined with weight training significantly contribute to aerobic capacity, increased metabolic efficiency, as well as low BFP and increased muscle mass (Russel et al., 1998; Mikulić, 2008). Body fat expressed as both fat mass and fat percentage tends to be lower in juniors than in elite and sub-elite seniors, although it appears that BFP also decreases in elite rowers (Mikulić, 2008).

### Physiological characteristics

The identified correlations indicate that Power_peak_ [W] and ERG_rel_ VO_2max_ [L/min] were highly correlated with the time taken to complete the 2000-m rowing ergometer test by both women and men. These parameters were not considered in the partial correlation analysis presented in the Results section, but they will be discussed in this section because they considerably influence rowing performance (Jurišić et al., 2014; Egan-Shuttler et al., 2019). The results of basic analyses confirmed the well-known fact that an increase in total rowing power and VO_2max_ expressed in L/min shortens the time to complete a 2000-m rowing ergometer test by both females (Battista et al., 2007; Klusiewicz et al., 2014; De Larochelambert et al., 2020) and males (Russel et al., 1998; Yoshiga and Higuchi, 2000; Fell and Gaffney, 2001; Ingham et al., 2002; Lawton et al., 2013). However, special attention should be paid to the relationships that emerged after the effects of the remaining variables had been eliminated in a partial correlation analysis. In both sexes, the time of completing the distance of 2000 m was most significantly shortened by an increase in Force_max_ and RP_max_ (relative maximal power). Rowing time was also significantly shortened by an increase in relative peak power in males and an increase in ERG_rel_ VO_2max_ (in mL/min/kg) in females.

These findings indicate that relative values and their relationships with rowing performance should be also considered in statistical analyses to facilitate the selection of athletes with the optimal and relatively lowest BFP. Most research conducted on rowers analyzed absolute values, but the significance of relative peak power (Alfoldi et al., 2021; Podstawski et al., 2022) and realative VO_2max_ (Mikulić, 2008; Alfoldi et al., 2021; Almeida-Neto et al., 2022; Papadakis et al., 2022; Podstawski et al., 2022) is increasingly recognized. During progressive exercise, individuals with higher BFP are characterized by higher oxygen demand, more rapid and shallow breathing, higher ventilatory demand, but a lower anaerobic threshold than athletes with higher lean body mass (Li et al., 2001; Vargas et al., 2018). As a result, lean body mass is closely related with the aerobic and anaerobic capacity of rowers (Durkalec-Michalski et al., 2019), which may be demonstrated during successive races in a regatta. During the finals, the performance of a rower with a higher BFP can decrease significantly relative to the results achieved during the qualifications. The phases of the rowing cycle and the differences between aerobic and anaerobic metabolism also play an important role in analyses of 2000-m rowing performance because these factors affect the production of cellular energy during physical effort. During the start, athletes use high stroke rates to increase the vessel’s displacement speed. Thus, speed is achieved and maintained mainly with the energy generated by the glycolytic pathway (Martin and Tomescu, 2017). During the central phase, the stroke rate decreases proportionally to the metabolic rate; the supply of energy from the glycolytic pathway is reduced, and most of the supplied energy is generated by aerobic pathways (between 65% and 75%). In the finishing phase, the stroke rate increases, and most energy is produced by anaerobic metabolic pathways (Held et al., 2020). Regardless of age and gender, total body mass and motor fitness levels can promote changes in the efficiency of cellular energy production in the human body (McNarry and Barker, 2018). However, biological maturation processes peak during adolescence, and the functioning of biological systems, including musculoskeletal and metabolic systems, is optimized during that period (Scheffler and Hermanussen, 2018). Therefore, the biological age is not always in sync with the chronological age and may be delayed or advanced (Moore et al., 2015). In metabolic terms, the efficiency with which cellular energy is produced by the oxidative pathway during physical exercise increases in advanced stages of biological maturation (Rowland, 2008; Ratel and Blazevich, 2017).

Almeida-Neto et al. (2022) found that advanced biological maturation in young elite rowers increased anaerobic power and decreased the time to complete the 2000-m rowing test both indoors and during competitive events. Dantas et al. (2018) found a significant relationship between the advancement of biological maturation and increased upper and lower limb power in rowers of both sexes. In the present study, higher chronological age was associated with a significantly shorter time of completing the 2000-m rowing test because the analysis involved both female and male rowers from different age groups (juniors to seniors).

## Strengths and limitations

One of the strengths of this study was that a partial correlation analysis was used to identify the APCh that exert the greatest impact on the performance of young rowers during a 2000-m rowing ergometer test. The relatively small number of senior female rowers was a potential limitation. Mikulić (2008) observed that the collection of extensive data on well-trained subjects is seldom practical or possible due to the limited access to such subjects. Moreover, performance measurements were obtained in a controlled laboratory environment, and it is debatable whether a maximal performance test can be carried out on a rowing ergometer, and whether the results acquired in different age groups can be used to predict real-world ergometer performance (Mikulić and Ružić, 2008; Almeida-Neto et al., 2020). In the present study, only the participants’ chronological age was considered, but the correlations between biological age and the time to complete the 2000-m rowing ergometer test were not determined.

## Conclusion

The results of this study, which involved a partial correlation analysis, suggest that the relative values of motor fitness indicators should be taken into account when describing the physiological profiles of rowers. The performance of both male and female rowers, including junior athletes, is not determined by a single factor, but several variables. Research has shown that rowing performance over a distance of 2000 m is significantly correlated by SMM, maximal force, relative maximal power, relative peak power, and estimated relative maximal aerobic capacity. In males, rowing time also decreased significantly with an increase in age, which confirms that older and more experienced athletes are more successful than juniors.

## Data Availability

The raw data supporting the conclusion of this article will be made available by the authors, without undue reservation.
